# Magnetic nanoparticles: a strategy to target the choroidal layer in the posterior segment of the eye

**DOI:** 10.1038/srep43092

**Published:** 2017-03-03

**Authors:** Martina Giannaccini, Lucia Pedicini, Guglielma De Matienzo, Federica Chiellini, Luciana Dente, Vittoria Raffa

**Affiliations:** 1 Department of Biology, Università di Pisa, 56127, Pisa, Italy; 2 Department of Chemistry and Industrial Chemistry, Università di Pisa, 56124, Pisa, Italy; 3 Institute of Life Science, Scuola Superiore Sant’Anna, 56127, Pisa, Italy

## Abstract

Despite the higher rate of blindness due to population aging, minimally invasive and selective drug delivery to the eye still remains an open challenge, especially in the posterior segment. The retina, the retinal pigment epithelium (RPE) and the choroid are posterior segment cell layers, which may be affected by several diseases. In particular, damages to the choroid are associated with poor prognosis in the most severe pathologies. A drug delivery approach, able to target the choroid, is still missing. Recently, we demonstrated that intravitreally injected magnetic nanoparticles (MNP) are able to rapidly and persistently localise within the RPE in an autonomous manner. In this work we functionalised the MNP surface with the vascular endothelial growth factor, a bioactive molecule capable of transcytosis from the RPE towards more posterior layers. Such functionalisation successfully addressed the MNPs to the choroid, while MNP functionalised with a control polypeptide (poly-L-lysine) showed the same localisation pattern of the naked MNP particles. These data suggest that the combination of MNP with different bioactive molecules could represent a powerful strategy for cell-specific targeting of the eye posterior segment.

The eye displays a very fine architecture organised in different cell layers, each one deputed to specific functions. It is composed of two segments enveloped by the sclera: the anterior segment, including the cornea, the aqueous humour and the iris and the posterior segment, containing the lens, the vitreous humour, the neural retina, the retinal pigment epithelium (RPE) and the choroid. The choroid is mostly composed of blood vessels, being one of the most vascularised tissues in the human body with the highest blood flow^1^. It has multiple crucial roles: accomplishment to the trophic sustainment of the RPE and, subsequently, of the photoreceptors; drainage of the aqueous humour from the anterior segment; thermoregulation of the eye; secretion of growth factors and refinement of the retina position by changing the choroid thickness^1^.

The major diseases affecting the posterior eye segment, such as age-related macular degeneration (AMD)^2^, diabetic macular edema (DME)^3^, proliferative vitreoretinopathy (PVR)^4^, uveitis^5^ and cytomegalovirus (CMV) retinitis^6^, affect different ocular structures. Non-exudative AMD breaks down RPE and photoreceptors. Exudative AMD causes growth of abnormal blood vessels behind retina and macula, disruption of Bruch’s membrane and degeneration of RPE^2^. DME is characterised by aberrations in retinal blood vessels. PVR and CMV lead to an inflammation of the retina, while uveitis is an inflammation of the uvea. However, whenever choroid alteration occurs, it leads to a poor prognosis^7^. It has been also suggested that the retina, the RPE and the choroid should be alternative targets in the treatment of different pathologies, and, in principle, their global and unspecific exposure to the therapeutic agent could raise side effects^8^. Ideally, a drug delivery system should be conceived to target the specific cell type, avoiding unspecific exposure of other cell types to the medication.

Preferred routes of drug administration for the treatment of the pathologies of the posterior segment are injections, both intravitreal (IVT) and subretinal. Drug instillation (as drops or gels) and systemic administration are often ineffective to reach the posterior segment, because of the poor cornea/sclera permeability and of the blood retinal barrier, respectively. Subretinal injections require high experience of the operator, with a concrete risk of retinal detachment. Intravitreal injections lack of any specificity, with drugs solely available at tissues surrounding the vitreous with an unfavourable kinetics (i.e., initial drug burst, rapidly declining over the time). The present work would contribute to the development of a novel nanoformulation for IVT injection with capability to preferentially release its cargo into the choroid layer. The animal model used in this study was zebrafish at the larval stage 0–120 hours post fertilisation (hpf). In zebrafish, the IVT injection is routinely used for delivering drugs to the posterior segment, e.g., antioxidants^9^ and growth factors^10^. Similarly to all vertebrates, fishes and humans eyes share the same anatomical structure, with the same neuronal architecture composed of five types of neurons (ganglion cells, horizontal cells, amacrine cells, bipolar cells and photoreceptors) arranged in three specific layers (ganglion, inner and outer nuclear layer)^11^,^12^. Most important, similarities extend beyond morphology with conserved gene expression and molecular pathways^13^,^14^,^15^, displaying closely related phenotypes in humans and zebrafish alike^15^,^16^,^17^. These similarities account for the frequent use of zebrafish as a model of human eye disorders over the last 10 years. Several models of ocular human diseases produced in zebrafish are currently used in research, exploiting natural or induced mutations^18^,^19^ and physical damage induction^20^,^21^,^22^. Among others, specific models of AMD recapitulate the physiopathology of the human disease, e.g. the *gnn* mutant displaying dystrophic cones and altered RPE, leading to loss of vision^23^.

In a previous work, we demonstrated in zebrafish embryos that IVT injected magnetic nanoparticles (MNP) are able to self-localise specifically to the RPE, without triggering any ocular toxicity^24^. We demonstrated that the localised particles persist over the weeks in the target tissue, leading to the possibility to have a long sustained drug release at the right location. This observation was in excellent agreement with several studies, reporting similar observations with other particles in other model systems^25^,^26^,^27^,^28^,^29^,^30^,^31^. The present work documents how the modification of the MNP surface with a bioactive molecule can change the fate of the particles and drive preferential accumulation in the choroid. Specifically, we selected the vascular endothelial growth factor (VEGF) as moiety for choroid targeting. Naked MNP and MNP functionalised with poly-L-lysine (PLL) were used as control groups. We observed distinct localisation patterns, with VEGF-bonded MNP preferentially localised in choroid and MNP or PLL-bonded MNP preferentially localised in the RPE.

## Results

### Particle functionalisation and characterisation

Carboxylic acid-stabilised iron oxide nanoparticles were covalently linked (peptide bond) to recombinant VEGF (hereafter labelled as rVEGF) or PLL via EDAC chemistry, according to a protocol we already published^32^. rVEGF was produced as a recombinant protein, with VEGF_165_ fused to the carboxy-terminus of glutathione-S-transferase (GST)^33^. It has been previously shown that the presence of GST in the fusion protein does not affect the biofunctionality of VEGF^33^. Actually, the presence of a GST tag facilitates the purification steps and works as a “spacer” for linking the VEGF to the MNP. The surface charge and hydrodynamics radius of the naked nanoparticles and the newly synthesised nanoparticles (hereafter labelled as MNP-rVEGF and MNP-PLL) were analysed. Naked MNP exhibit a negative Z potential (−28 ± 2 mV) and a hydrodynamic size of 61.0 ± 1.87 nm, which are in good agreement with the information provided by the supplier. However, their polydispersion index (PI) is higher than the value provided in the datasheet, being 0.26 ± 0.04, which suggests that particles are monodispersed (the 50% of the particle distribution was found to have a size lower than D50 = 69 ± 2.01 nm) or in small aggregates of 2–3 particles (the 90% of the particle distribution was found to have a size lower than D90 = 179.83 ± 14.06). After covalent binding of rVEGF, we found about 0.2 mg of recombinant protein per mg of MNP, which ideally correspond to 880 molecules exposed by each particle. The superficial charge of MNP-rVEGF was found to be more negative (−34.7 ± 2.21 mV), because of the isoelectric point (5.03) of the recombinant protein (deduced from the amino acid sequence). The increase of size (from 61.0 ± 1.87 to 128.57 ± 0.35 nm) is likely due to the presence of small aggregates of 2 particles (the size of the recombinant protein is expected to be 2–3 nm). The polydispersion index does not further increase compared to the naked MNP, being 0.22 ± 0.04. MNP-PLL showed a positive charge according to the cationic nature of PLL, an increase of the hydrodynamics diameter (from 61.0 ± 1.87 to 299.17 ± 19.53 nm) and a slight increase of the PI (from 0.26 ± 0.04 to 0.29 ± 0.01) (Table 1, Supplementary Figures S1–5), which suggest that, MNP-PLL preferentially cluster in small aggregates of few (2–4) particles. In general, data of the polydispersion index confirm that the distributions are narrow and the small aggregates are not related to particle instability but to events of cross-linking (peptides carry carboxylic groups, which can be activated by the residual traces of EDC).

**Figure 1:**
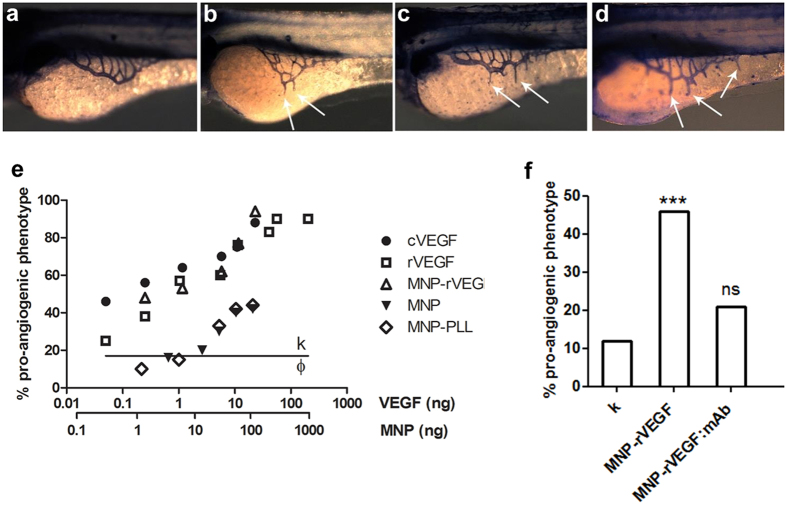
Neo-angiogenesis induction in SIV model. (**a**–**d**) Phosphatase alkaline staining. Larvae injected at 24 hpf and fixed at 72 hpf. (**a**) Normal phenotype. (**b**–**d**) Examples of neo-angiogenic phenotype induced by the injection of cVEGF, rVEGF and MNP-rVEGF, respectively. The white arrows point some of the extra vessels induced. (**e**) Dose-response curves of the neo-angiogenic phenotype. The black line corresponds to the percentage of the control experiments, i.e., embryos injected with saline (k, % = 18, n = 59) and GST (Φ, %19, n = 37). Regression analysis, exponential fit, 95% confidence level. 1-way ANOVA followed by Bonferroni correction: cVEGF vs rVEGF vs MNP-rVEGF and MNP vs MNP-PLL are not statistically significant, while all other combinations have p < 0.05. (**f**) Injection of MNP-rVEGF (0.5 ng of rVEGF) alone or complexed with bevacizumab. Chi-square test. n = 65 each group.

Binding of a protein to the particle surface cannot guarantee that the protein preserves its biofunctionality; therefore, functional assays are required. We exploited the angiogenic assay of subintestinal vein (SIV) in zebrafish embryos^34^ to compare the bioactivity of rVEGF and MNP-rVEGF. The SIV plexus of zebrafish embryos is an excellent system for studying neo-angiogenesis induction. At 72 hours post fertilisation (hpf), the SIV is organised in a clear defined basket-like shape that extends on the dorsolateral surface of the yolk on both sides. The precise ramifications can be easily marked by alkaline phosphatase staining, because the underneath yolk is an acellular region. This model is the elective model to study angiogenesis: it is very sensible and also weak pro-angiogenic stimuli result in a change of the phenotype, with new branch(es) sprouting from the basket and/or alterations in the basket architecture (supernumerary vessels and irregular formation). We injected equivalent amounts of VEGF into the yolk of zebrafish embryos at 24 hpf and two days later we analysed the shape and the number of SIV vessels (Fig. 1). Both rVEGF and MNP-rVEGF were able to induce a pro-angiogenic response in the SIV, comparable to commercial human VEGF (hereafter labelled as cVEGF) used as a positive control (Fig. 1a–f), thus confirming the bioactivity of rVEGF and its retention in MNP-rVEGF. In conclusion, neither the production of the recombinant protein nor its binding to the MNP alter the VEGF biofunctionality. Additionally, no significant mortality or malformations were observed compared to sibling controls.

As a further proof of the full retention of the neo-angiogenic potential of MNP-rVEGF, we further evaluated the dose-response of cVEGF, rVEGF and MNP-rVEGF (Fig. 1e). We injected also MNP and MNP-PLL that do not harbor any angiogenic stimulus. Other control groups included the saline solution and the GST alone expressed in plasmid-transformed bacteria (in order to exclude a possible role of this recombinant fragment in the pro-angiogenic activity). The incidence of the angiogenic phenotype was 18% for both saline solution (n = 59) and GST group (n = 37), corresponding to the physiological value found in non-injected embryos (12–18%, n = 50). The dose-response curve of rVEGF and MNP-rVEGF shows a substantial similarity, while the dose-response curve of cVEGF is sharper, suggesting that the commercial VEGF carries a stronger angiogenic potential. These results are not surprising, because western blotting analysis of the purified recombinant proteins revealed the presence of degradation products, which could differently contribute to the angiogenic potential, decreasing the efficacy per unit of mass (Supplementary Figure S6). We fitted the experimental data (see Material & Methods) to calculate the ED50, i.e. the effective dose inducing a 50% incidence of the neo-angiogenic phenotype. The ED50 obtained for cVEGF is in agreement with previous data in the literature^34^,^35^. MNP-rVEGF and rVEGF show a very similar ED50, even though a little higher proangiogenic activity for MNP-rVEGF was observed (it is likely that MNP protect rVEGF from degradation) (Fig. 1e, Table 2). Naked MNP and MNP-PLL do not show any proangiogenic activity, except in very high doses of particles, which we interpreted as a sign of particle toxicity (Table 2). This could be due to a toxic stress induced by the high concentration of free carboxylic and free amino groups exposed on the surface of MNP and MNP-PLL, respectively. However, the ED50 for MNP and MNP-PLL increases a hundredfold compared to that of MNP-rVEGF (2.91, 271.22 and 205.77 ng of MNP for MNP-rVEGF, MNP and MNP-PLL, respectively) (Table 2).

As a further proof of evidence, we evaluated if the treatment with an anti-VEGF antibody impairs the MNP-rVEGF mediated neoangiogenesis. We used a clinically employed anti-VEGF monoclonal antibody, bevacizumab (Avastin^®^), a humanised recombinant monoclonal immunoglobulin G (mAb). The injection of MNP-rVEGF:mAb in the SIV of zebrafish embryos leads to quasi-physiological levels of incidence of the angiogenic phenotype, as outlined in Fig. 1f.

### Particle localisation in the posterior eye segment

**Figure 2:**
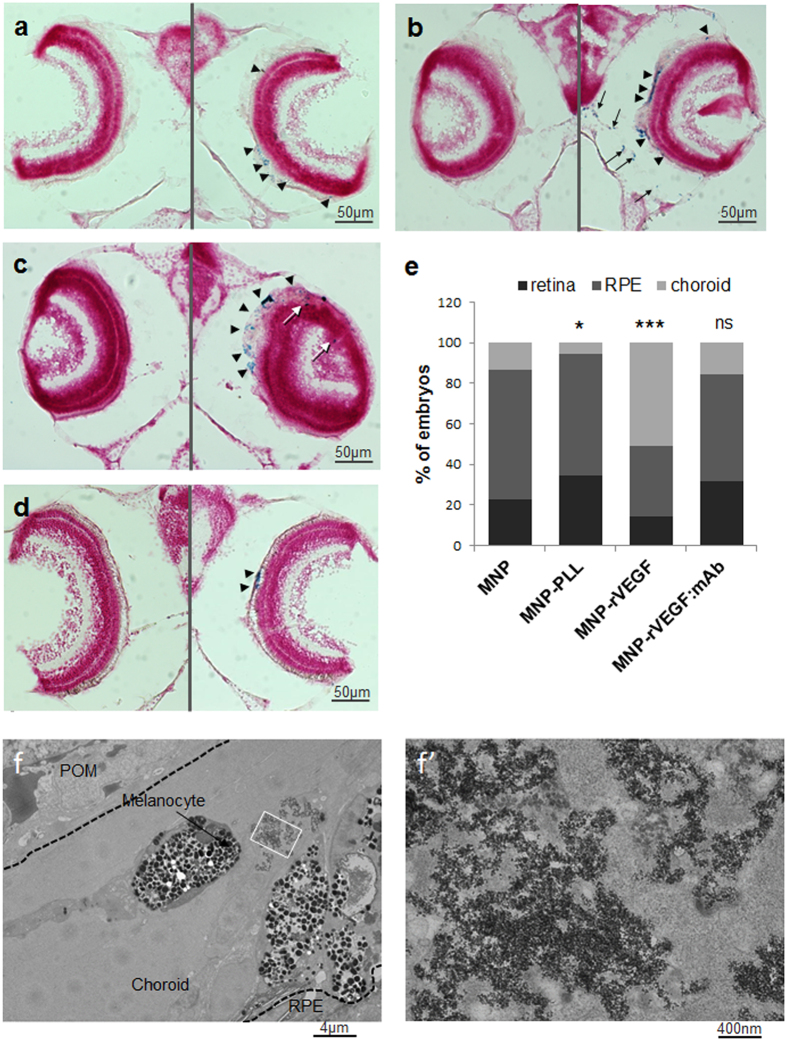
Larvae injected with particles at 48 hpf, and fixed at 24 hpi. Prussian blue staining on paraffin sections of embryos injected with (**a**) MNP, (**b**) MNP-rVEGF, (**c**) MNP-PLL and (**d**) MNP-rVEGF:mAb. Left: control side; right: injected side. Nanoparticles (blue stained) are pointed by arrowheads in the RPE, black arrows in the choroid and white arrows in the retina. (**e**) Percentage of embryos showing preferential particle localisation in retina, RPE or choroid. n = 45. Chi-square test. (**f**) TEM imaging of larvae injected with MNP-rVEGF. POM: periocular mesenchyme. (f’) Magnification of inset in F showing particle clusters.

Naked MNP, MNP-PLL, MNP-rVEGF and MNP-rVEGF:mAb were injected into the eye. Briefly, we injected 2 nl of the particles, near the lens of 72 hpf zebrafish embryos, using a pressure microinjector. After one day, we fixed the embryos and analysed the distribution of the particles inside the ocular structures via Prussian blue staining. The naked MNP localise efficiently in the RPE layer (Fig. 2a, arrowheads), as we previously demonstrated for other naked MNP^24^. Few particles can be also found in the retina. Similarly, MNP-PLL were found to localise in the RPE and the retina (Fig. 2c, white arrows). In contrast, if functionalised with the rVEGF, the MNP are able to cross the RPE layer and to localise in the developing choroid (Fig. 2b, black arrows). We deduced that localisation in the developing choroidal layer is driven by the molecule-specific signal carried out by MNP-rVEGF, which is supported by several experimental evidences. Actually, MNP-PLL are not able to reach the choroid, showing accumulation only in the retina and in the RPE (Fig. 2c, white arrow and black arrowheads, respectively), suggesting that the rVEGF guides the localisation of MNP in the choroid. Moreover, if the VEGF activity is blocked with bevacizumab, the MNP-rVEGF:mAb are not able to cross the RPE, where they remain preferentially trapped (Fig. 2d). A potential effect of different particle size or surface charge in driving the choroid localisation should be excluded, according to results we already published^24^. A quantitative data analysis is reported in Fig. 2e, showing the incidence of embryos with preferential particle localisation in retina, RPE or choroid. Data analysis showed that the localisation pattern of MNP-rVEGF is statistically different from that one of naked MNP (p-value < 0.0001), showing an increase of embryos with preferential particle localisation in the choroid. On the contrary, if MNP-rVEGF are combined with the mAb (MNP-rVEGF:mAb) the pattern is identical (p = 0.183). Interestingly, MNP-PLL show a weak increase of embryos percentage with preferential particle localisation in retina compared to naked MNP (p = 0.036). The analysis of the ultrastructure was performed by transmission electron microscopy (TEM) in order to confirm the localisation of MNP-rVEGF in the choroid. TEM imaging allows easily recognising the choroid (Supplementary Figure S7). The MNP-rVEGF were found to localise in the choroid (Fig. 2f-f’), confirming the histological observation.

**Figure 3:**
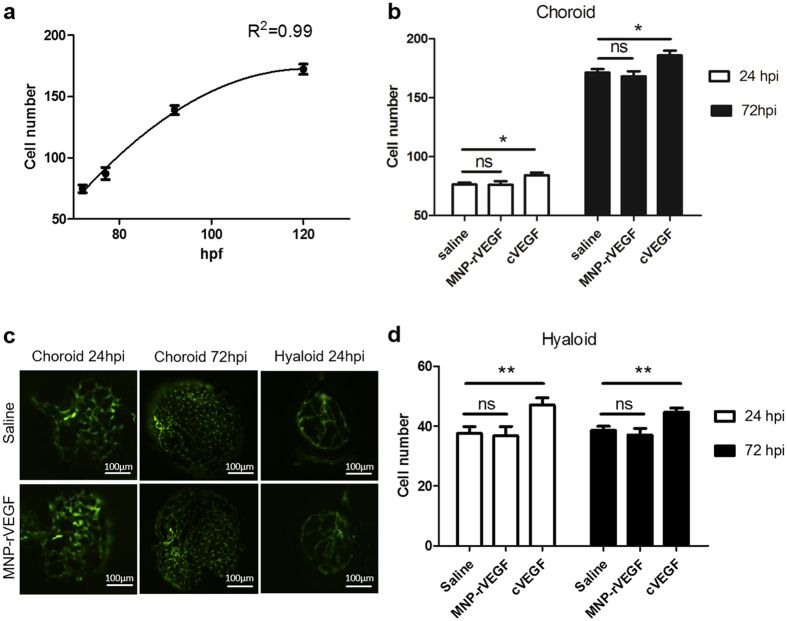
(**a**) Choroid of eyes explanted from transgenic zebrafish with fluorescent vessels. The number of choroidal endothelial cells correlates with developmental angiogenic stimulus. n = 12. (**b**) Quantification of endothelial cells in the choroid of larvae injected at 48 hpf with saline or MNP-rVEGF or cVEGF and fixed at 24 hpi or 72 hpi. n = 11. Statistical analyses were performed by 2-way ANOVA followed by Bonferroni correction. MNP-rVEGF vs control are not statistically significant, while cVEGF vs control has p = 0.018 and p = 0.0035 at 24 and 72 hpi, respectively. (**c**) Representative images of the choroid and the hyaloids of larvae injected in the left side with MNP-rVEGF and in the right side with saline. Dorsal side on the top, anterior on the right. n ≥ 11. (**d**) Quantification of endothelial cells in the hyaloid of larvae injected at 48 hpf with saline or MNP-rVEGF or cVEGF, fixed at 24 or 72 hpi. n = 15. Statistical analyses were performed by 2-way ANOVA followed by Bonferroni correction. MNP-rVEGF vs control is not statistically significant, while cVEGF vs control has p = 0.004 and p = 0.0014 at 24 and 72 hpi, respectively.

Obviously, we considered that the specific localisation of MNP-rVEGF could also trigger an undesirable angiogenic stimulus in the choroid. Therefore, we took advantage of the use of transgenic zebrafish embryos with green vessels (roy-/-;nacre-/-;Tg(kdrl:egfp)^s483^)^36^ in order to verify this aspect. These embryos specifically express green fluorescent protein (GFP) in endothelial cells. Because a model to study angiogenesis in the choroid of zebrafish is missing in the literature, we exploited a method to follow spontaneous angiogenesis in developing embryos. We found that the number of endothelial cells, which constitute the choroid of larvae in the experimental window from 72 to 120 hpf, is an excellent parameter, with cell number increasing over the time (Fig. 3a). Later, endothelial cells start to organise in round shaped vessels and the anatomical location and the 3D structure of the vessels do not allow for a precise cell counting. By using this model, we found that MNP-rVEGF do not induce neo-angiogenesis (the number of endothelial cells in the injected eye is not significantly different from that in the non-injected control side, even at the last time point tested (Fig. 3b,c). On the contrary, the same amount of cVEGF (positive control) triggered a statistically significant angiogenic response (p = 0.013 and p = 0.009 at 24 and 72 hpi, respectively) (Fig. 3b).

We further tested the hyaloid, which is the only other vascular plexus of the eye posterior segment at these development stages (0–120 hpf) in order to exclude any other undesired angiogenic effect. Similarly, we counted the number of hyaloid endothelial cells and we found that MNP-rVEGF do not increase the cell number with respect to the control eye (p = 0.44 and p = 0.25 at 24 and 72 hpi, respectively), in contrast to cVEGF (p = 0.004 and p = 0.0014 at 24 and 72 hpi, respectively) (Fig. 3c,d).

## Discussion

The efficient, specific and controlled drug delivery in the posterior segment of the eye still remains a challenge. The eye has physical barriers rendering it poorly permeable to both locally and systemically administered drugs. Indeed, the cornea/sclera barriers prevent locally instilled drugs from reaching the posterior segment. At the same time, a blood retinal barrier prevents drug penetration inside the eye from the blood stream. The only effective way to reach the posterior segment, and specifically the retina, the RPE and the choroid, is via intravitreal or subretinal injections. Subretinal injections are more invasive then intravitreal ones, but both of them require repetitive injections because of the short half-life of drugs and poor retention. An additional drawback of intravitreal injections is the lack of specificity; in fact, tissues in contact with the vitreous, without distinctions, are exposed to the drug. Recently, nanomedicine opened new perspectives, offering novel nanocarriers for an efficient drug delivery^37^. It was largely demonstrated that nanomaterials increase the half-life of drugs^38^. Concerning the ocular drug delivery, nanocarriers offer new opportunities for controlling the localisation of the release and its kinetics^39^,^40^ and to perform therapies such as gene therapy^41^ and laser therapy^42^. In a previous work we demonstrated that magnetic iron oxide nanoparticles are able of self-accumulation in the RPE^24^. MNP are biodegradable (entering in the normal iron metabolism)^43^,^44^, intrinsically safe and there are different iron oxide based nanoformulations already approved for use on humans^45^,^46^ e.g., Combidex^®^ (MRI contrast agent for differentiation of metastatic and non-metastatic lymph nodes), Endorem^®^ (MRI contrast agent for diagnosis of liver tumors), Resovist^®^ (MRI contrast agent for diagnosis of liver metastases and colon cancer), Feraheme^®^ (indicated for the treatment of iron deficiency anemia in adult patients with chronic kidney disease). Specifically, there are evidences from studies in rats that the iron oxide MNPs are non-toxic to the ocular structures^47^. MNP show other interesting features beyond the pure carrier capability, such as acting as contrast agent in MRI imaging^48^, in magnetic hyperthermia^49^ and being long distance controlled by magnetic fields^50^.

**Figure 4:**
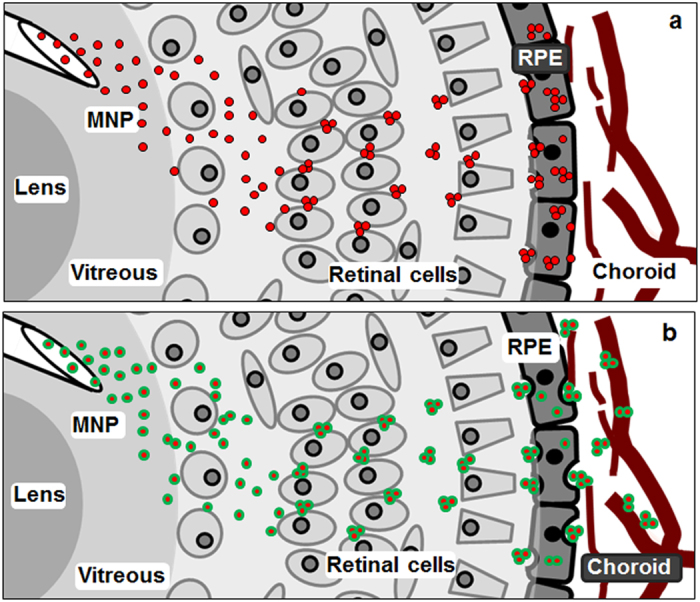
Model for MNP localisation. (**a**) Naked MNP or MNP-PLL localise in the RPE; after injection, particles migrate towards the narrow intercellular spaces of retinal cells and their crossing induces the formation of clusters, which are easily phagocyted by the RPE. (**b**) MNP-rVEGF are able to localise in the choroid; they probably escape from RPE through transcytosis, moving towards the choroid where they are retained, likely due to VEGF receptor binding.

Figure 4 describes the extension of a model we previously proposed to explain the autonomous localisation of MNP into RPE^51^. The MNP are small enough to diffuse into the vitreous and among the retinal cell layers, where the particles would be forced to move along a narrow intercellular space, causing their clusterisation due to dipole-dipole interaction among magnetic particles. In this way, the particles could reach the RPE as small aggregates with a micrometric dimension ideal for promoting engulfment by RPE^51^ (Fig. 4a). In the present paper we investigated if surface functionalisation with biomolecules could change the fate of MNP, driving their localisation in the choroid. Specifically, we used a recombinant VEGF as a targeting moiety. According to the current knowledge, we hypothesise that, once internalised by RPE, the rVEGF could induce particle transcytosis towards the choroid (Fig. 4b). In fact, it is known that RPE cells normally secrete VEGF, which is exocytosed to sustain the growth and the polarised fenestration of choroidal vessels during development^52^,^53^. Most important, exogenous VEGF was found to cross the RPE monolayer by transcytosis, predominantly in the apical-to-basal direction^54^. Moreover, the RPE has mechanisms for maintaining low concentrations of VEGF in the retinal space, including VEGF endocytosis^52^. VEGF could thus offer an additional stimulus in promoting particle internalisation by the RPE.

In the present work, we produced a human recombinant VEGF, which was covalently attached to MNP. We characterised the functionalised nanoparticles demonstrating *in vivo* that the rVEGF biofunctionality is not lost in the process of particle functionalisation (Fig. 1). As control group, we choose to functionalise the MNP with a non-bioactive peptidic molecule, i.e. poly-L-lysine. All samples appeared to be monodispersed particles or submicrometric clusters (Table 1).

We found that the functionalisation of MNP with rVEGF effectively triggers preferential accumulation in the choroid (Fig. 2). This evidence supports the idea that rVEGF stimulates particle transcytosis across the RPE, similarly to the endogenously produced VEGF during physiological secretion processes. Once reached the choroid, particles are likely retained by endothelial cells, which expose the VEGF receptor 2, sequestering them (Fig. 4b). According to this idea, we explored if the inactivation or the sequestration of VEGF impairs the observed mechanism. VEGF was hampered by the monoclonal antibody bevacizumab. Bevacizumab binds human VEGF with high affinity (Kd ≈ 0.5 nM) and structural analysis of VEGF bound to bevacizumab suggested that the mAb is effective by sterically disrupting the ability of VEGF to interact with its receptors^55^. Experimental data confirmed that, after complexation of MNP-rVEGF with bevacizumab, the particles completely lost their ability to pass the RPE and to localise in the choroid, suggesting an active role of the VEGF in promoting the localisation of the particles in the choroid. This finding is further supported by the observation that poly-L-lysine coated MNP do not pass the RPE, displaying preferential accumulation in the retina and in the RPE. It is worth to mention that particle size or superficial charge do not play a role in driving localisation^24^. Here we confirmed previous finding, showing that MNP and MNP-rVEGF which have similar Z potential, show a totally different localisation while MNP and MNP-PLL, which have different size, exhibit a similar localisation pattern. Conversely, the bioactivity of the molecules bonded to the particle surface is likely the only influencing factor. According to this, the inhibition of the bioactivity of the exposed molecule (in our case by using a monoclonal antibody) caused a full recovery of the typical localisation profile of naked MNP (i.e. RPE localisation).

However, the localisation of an angiogenic stimulus in the choroid could have serious side effects. Therefore, since the neovascularisation of the choroid is an unwanted side effect, we tested if the localisation of the MNP-rVEGF in the choroid triggers a pro-angiogenic stimulus. The results obtained by IVT injecting 0.7 ng rVEGF suggest that even if the levels of rVEGF locally reached are sufficient to drive preferential localisation in the choroid (acting as a targeting moiety), they do not to induce local activation of the neo-angiogenesis.

Our work outlines that the functionalisation of particles with biomolecules may lead to different particle localisation. We speculate that this finding could be exploited to design novel carriers for cell-specific ocular targeting, in particular for the choroid. Once reached the desired target, the functionalised MNP could be used to deliver their cargo with the desired kinetics profile. Their long half-life could sustain drug delivery over weeks or months. According to our findings, VEGF conjugation to MNP could be used to specifically target the choroid. In order to further improve the safety of MNP-VEGF as a drug carrier, VEGF could be easily replaced with a mutant protein (e.g., a mutant which is able to bind the receptor, without inducing receptor dimerisation^56^ and thus neo-angiogenesis) or an analogue able to trigger particle translocation, but not the activation of the neo-angiogenic pathway. Obviously, the choroid is a very interesting target. Many diseases cause neovascularisation of choroidal vessels leading to bad prognosis, such as AMD (the wet form)^57^, myopic macular degeneration^58^, uveitis^59^, presumed ocular histoplasmosis syndrome^60^, and angioid streaks^61^. It is obvious that the prevention or reduction of neovascularisation is the first aim in the treatment of degenerative ocular diseases; however, a specific drug targeting to the choroid is missing. In such context, properly functionalised particles could be used to deliver small inhibitors of angiogenesis or any other therapeutics for the treatment of choroid-related diseases, specifically targeting the choroid with a controlled release and a long persistence, without side effects for the retinal vasculature.

## Conclusions

The posterior eye segment may be affected by several diseases, which account for the majority of blindness worldwide. It is composed of different tissues and each one can be a crucial therapeutic target, depending on the pathology or the stage of the disease. However, all together, unspecific targeting and poor drug retention can lead to dangerous side effects. Currently, tissue-specific ocular delivery of therapeutic drugs still remains a utopia. In this context, the present work demonstrates that the covalent functionalisation of MNP with a recombinant VEGF changes the localisation fate of intravitreal injected MNP, preferentially driving them to the choroid, while naked MNP or poly-L-lysine coated MNP account for preferential localisation in the RPE. These findings open new possibilities for the exploitation of MNP as ocular drug carriers, conferring them the capability of selective targeting of distinct ocular tissues in the posterior eye segment.

## Methods

### Production of the recombinant VEGF

The coding region of human VEGF_165_ isoform (gift from Dr. Gilda Cobellis) was subcloned in pGEX6p-1 plasmid in order to express in bacteria the recombinant protein VEGF_165_ fused at the carboxy-terminus of glutathione-S-transferase (GST). We used GST alone produced from bacteria transformed with the empty pGEX6p-1 plasmid as control. For protein purification we followed the protocol published by Morera and colleagues^33^ with minor modifications. In particular, we transformed BL21 strain of *E. coli* with pGEX6P-VEGF and a single colony was inoculated in 10 ml LB medium. After overnight growth, it was transferred into in 1 litre of LB medium containing 100 μg/ml ampicillin. Recombinant gene expression was induced with IPTG at final concentration of 1 mM for 4 hours at 37 °C in a shaker. Bacterial cells were centrifuged at 2000 g and lysed (lysozyme 0.2 μg/ml, DTT 10 mM, protease inhibitors, triton x-100 1%, MgCl_2_ 10 mM and DNAse 0.1 mg/ml). The lysed cells were centrifuged at 20000 g and the supernatant was purified adding Glutathione-Sepharose 4B resin (resin/lysate ratio 1:1333) according to manufacturer’s instructions. After resin washing with PBS, the elution was performed by adding 50 mM Tris/HCl and 10 mM GSH, pH 8. The eluate was dialysed against PBS and concentrated (1:3) using ultrafiltration spin columns. Protein concentration was calculated by spectrophotometric determination at 280 nm (using a calibration curve). The yield of recombinant VEGF fusion protein was about 2 mg from 1 litre of bacterial growth.

### Particle functionalisation

Commercial MNP produced by Micromod (79-02-501, nanomag® -D –spio) were used. According to the datasheet, MNP have a magnetite core of iron oxide (50 nm in size), an organic shell which exposes -COOH groups, a mean hydrodynamic particle diameter in the range of 60–100 nm, a polydispersity index < 0.2 and a magnetisation of 24 emu/g particles (H = 1000 Oe). Particles were sonicated before the use. An electron microscopy image of the sample is shown in Supplementary Figure S1.

Particles were covalently functionalised with rVEGF or poly-L-lysine (70,000–150,000 Da) via EDAC chemistry. Briefly, 1 mg of particles was ultracentrifuged (18000 g) and resuspended in 500 μl of a 10% EDAC water solution. After ten minutes, the protein was added (protein:MNP ratio was: 3.51 w/w) and mixed for 1 hours at 4–8 °C. The unbounded protein was removed by ultracentrifugation (18000 g) and by discharging the supernatant (two washing steps). The nanoparticles were suspended in a 20% glycerol water solution and aliquots were stored at −20 °C. The amount of protein bound to the surface of MNP was calculated by subtraction, i.e., by measuring the absorbance at 280 nm of the supernatant derived from the washing steps. The protein concentration was obtained by using a calibration curve obtained with a known amount of protein. The amount of MNP was assessed by thiocyanate colorimetry. Briefly, the samples were incubated for 1 h at 60 °C in HCl 6 M: plus HNO_3_ 65%, then sample was water diluted 1:10 and an equal volume of KSCN 1.5 M was added. Absorbance at 478 nm was immediately recorded. Known concentrations of MNP were used to obtain a calibration curve.

The composition of MNP-rVEGF and MNP-PLL was calculated to be 1.8 mg/ml of MNP, 350 μg/ml of protein, 20% of glycerol.

The functionalised particles were characterised in terms of hydrodynamic size and Z-potential by Zeta sizer NanoTM (Malvern Instrument).

For the preparation of the complex MNP-rVEGF and bevacizumab, we incubated the MNP-rVEGF with bevacizumab in a molecular ratio of 1:50 for 15 minutes. The excess of bevacizumab was removed by magnetic separation and sample washing.

### Embryo preparation

Animal procedures were performed in strict compliance with protocols approved by Italian Ministry of Public Health and the local Ethical Committee of the University of Pisa (authorisation n. 99/2012-A, 19.04.2012), in conformity with the Directive 2010/63/EU. Zebrafish embryos (roy-/-; nacre-/-; tg(*kdrl:egfp*)^s483^) were obtained by natural mating and staged according to manuals^62^ (authorisation n° 1173/2015-PR). The embryos were anesthetised in 0.02% tricaine. If necessary, the chorion was manually removed at 24 hpf.

### Embryo microinjections in SIV region

30 nanolitres of naked MNP, MNP-rVEGF, rVEGF, cVEGF or MNP-rVEGF:mAb were microinjected in the yolk of anesthetised zebrafish at 24 hpf by using a pneumatic picopump injector. After injection, embryos were reared at 28 °C and were sacrificed at 72 hpf. Each experiment was replicated three times. Each replicate was performed at least on 15 larvae per group. The phenotype was considered “pro-angiogenic” if at least one supernumerary vessel was present.

### Embryo microinjections in the eye

The embryos were anesthetised and embedded in 0.3% agarose. Two nanolitres of MNP, MNP-PLL, MNP-rVEGF or MNP-rVEGF:mAb were microinjected in the left eye at 48 or 72 hpf. After injection, embryos were reared at 28 °C and sacrificed at 24, 29, 44 or 72 hpi. Each experiment was replicated three times. Each replicate was performed at least on 11 larvae per group.

### Histological analysis

Zebrafish embryos were fixed in 4% paraformaldehyde for 2 h, after which they were embedded in paraffin and sectioned (10 μm). The paraffin sections were stained by Prussian Blue according to the manufacturer’s instructions (Sigma-Aldrich, St. Louis, USA), after a treatment of pigment bleaching in 5% formamide-1% hydrogen peroxide in the presence of cold light. Images were captured with Nikon Eclipse E600 microscope.

Blood vessels of whole embryos were stained using alkaline phosphatase staining. After fixation in 4% paraformaldehyde for 2 h, embryos were gradually dehydrated in ethanol and rehydrated in alkaline buffer (Tris 0.1 M HCl pH 9.5, 50 mM MgCl2, 0.1 M NaCl, 0.1% Tween 20) for 30 minutes. Once the embryos equilibrated in alkaline buffer, NBT/BCIP was added. After staining for 10 min, all blood vessels in the fish were labelled. Embryos were imaged using a stereomicroscope Nikon SMZ1500.

For choroid and hyaloid imaging the larvae were fixed in 4% paraformaldehyde for 10 minutes and washed two times for 10 minutes in PBS. The eyes were explanted and the lens and epidermidis were carefully removed with forceps. The explants were mounted and pictures were immediately recorded with a Nikon Eclipse Ti microscope.

For transmission electron microscopy, the zebrafish larvae fixed for ultrastructural studies were trimmed and post-fixed with 2.5% glutaraldehyde and 2% osmium before embedding the tissue in Epon-araldite mixture. Thin sections were placed on copper grids and stained with uranyl acetate and lead citrate. All samples were examined under JEOL 100Xl TEM.

### Statistical analysis

Values are reported as the mean ± standard error of the mean. Significance was set at p ≤ 0.05. “*”^,^ “**”^,^ “***”^,^ “ns” are p < 0.05, p < 0.01, p < 0.001, not significant, respectively. Statistical analyses were performed by ANOVA followed by Bonferroni correction or Chi-square test.

For dose-response curve, data were fitted in Matlab R14 workspace with the exponential function


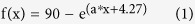


where x is the VEGF concentration, by fixing the confidence level at 95%. The equation was derived from imposing the following boundary conditions (from experimental data):


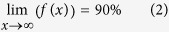



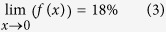


## Additional information

**How to cite this article:** Giannaccini, M. *et al*. Magnetic nanoparticles: a strategy to target the choroidal layer in the posterior segment of the eye. *Sci. Rep.* **7**, 43092; doi: 10.1038/srep43092 (2017).

**Publisher's note:** Springer Nature remains neutral with regard to jurisdictional claims in published maps and institutional affiliations.

## Supplementary Material

Supplementary Information

## Figures and Tables

**Table 1:** Z potential, diameter and polydispersion index (PI) of naked and functionalised nanoparticles.

	Z potential (mV)	Diameter (nm)	PI
MNP	−27.56 ± 1.85	61.0 ± 1.87	0.26 ± 0.04
MNP-rVEGF	−34.7 ± 2.21	128.57 ± 0.35	0.22 ± 0.04
MNP-PLL	+ 14.68 ± 0.90	299.17 ± 19.53	0.29 ± 0.01

n = 3. The full graphs are provided in Supplementary Figures S1–4.

**Table 2:** ED50 of cVEGF, rVEGF, naked and functionalised nanoparticles, based on data fitting of Fig. 1.

Sample	ED_50_		R square
	VEGF (ng)	MNP (ng)	
cVEGF	0.11		0.95
rVEGF	0.83		0.96
MNP-rVEGF	0.57	2.91	0.84
MNP		271.22	0.92
MNP-PLL		205.77	0.96

n = 30 each group.
