# Supplementary material for: Magnetic nanoparticles: a strategy to target the choroidal layer in the posterior segment of the eye
Source: Sci Rep. 2017 Mar 3;7:43092. doi: 10.1038/srep43092 (PMC5335660; doi:10.1038/srep43092)
Supplement: Supplementary Information [file srep43092-s1.pdf]

Magnetic nanoparticles: a strategy to target the choroidal layer in the posterior segment of the eye

Martina Giannaccini[1]*, Lucia Pedicini[1], Guglielma De Matienzo[1], Federica Chiellini[2], Luciana Dente[1], Vittoria Raffa[1,3]

1. Department of Biology, Università di Pisa, 56127, Pisa, Italy

2. Department of Chemistry and Industrial Chemistry, Università di Pisa, 56124, Pisa, Italy

3. Institute of Life Science, Scuola Superiore Sant'Anna, 56127, Pisa, Italy

*corresponding author: martina.giannaccini@gmail.com; phone +39 0502211537; fax +39 0502211495

Supplementary materials

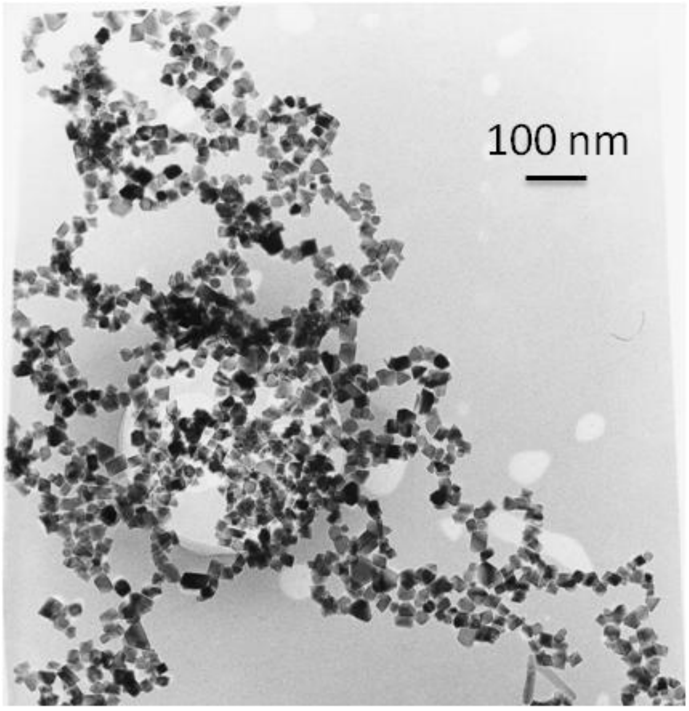

Figure S1: Commercial MNP produced by Micromod (79-02-501, nanomag® -D –spio)

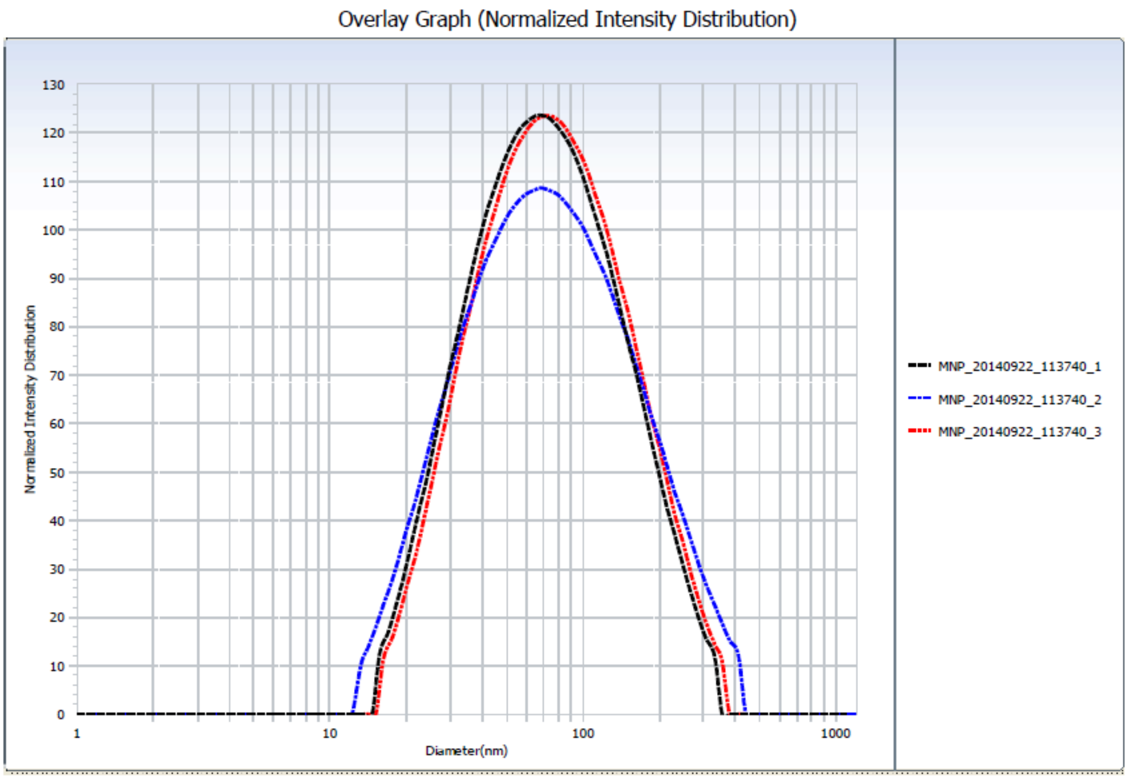

No	Data	Repet. No	pH	Ave. Diameter(nm)	Polydispersity Index	D (10%) (nm)	D (50%) (nm)	D (9
1	MNP_20140922_113740_1	1	NA	60.8	0.231	27.8	67.1	
2	MNP_20140922_113740_2	2	NA	59.4	0.309	25.3	68.8	
3	MNP_20140922_113740_3	3	NA	63.1	0.250	29.3	71.1	

Figure S2: DLS analysis of MNP samples

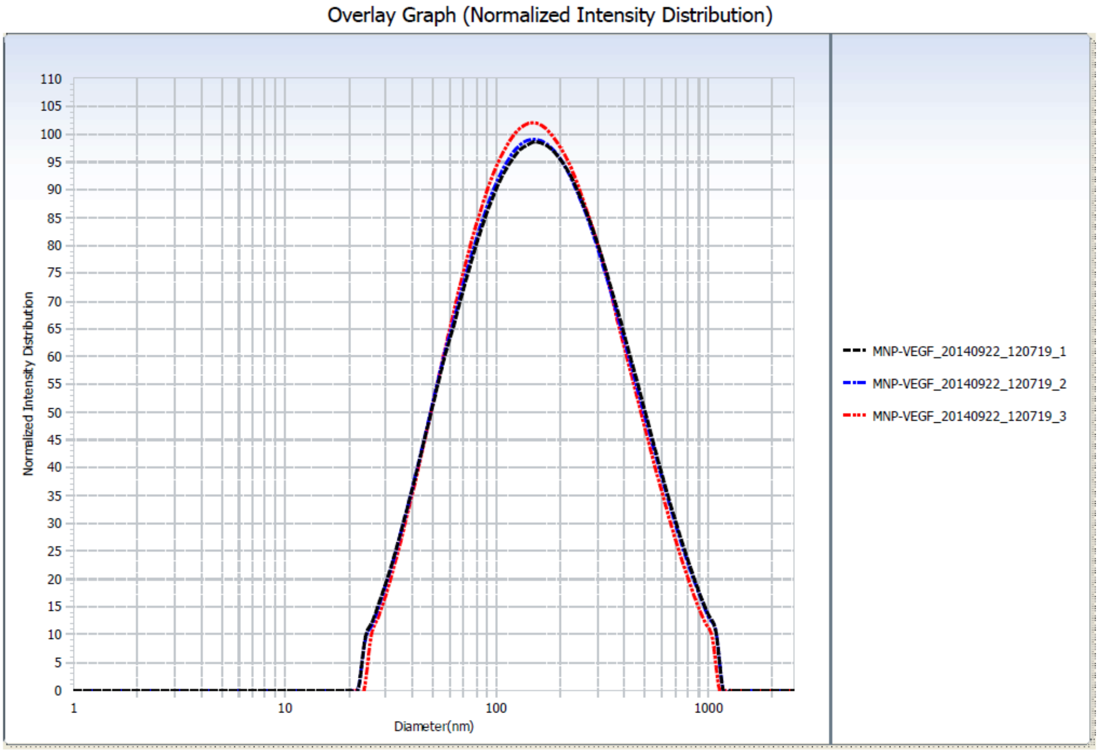

No	Data	Repet. No	pH	Ave. Diameter(nm)	Polydispersity Index	D (10%) (nm)	D (50%) (nm)	D (90%) (nm)
1	MNP-VEGF_20140922_120719_1	1	NA	128.9	0.302	49.8	151.4	475.7
2	MNP-VEGF_20140922_120719_2	2	NA	128.6	0.294	49.9	150.0	471.6
3	MNP-VEGF_20140922_120719_3	3	NA	128.2	0.286	51.0	148.2	452.2

Figure S3: DLS analysis of MNP-rVEGF samples

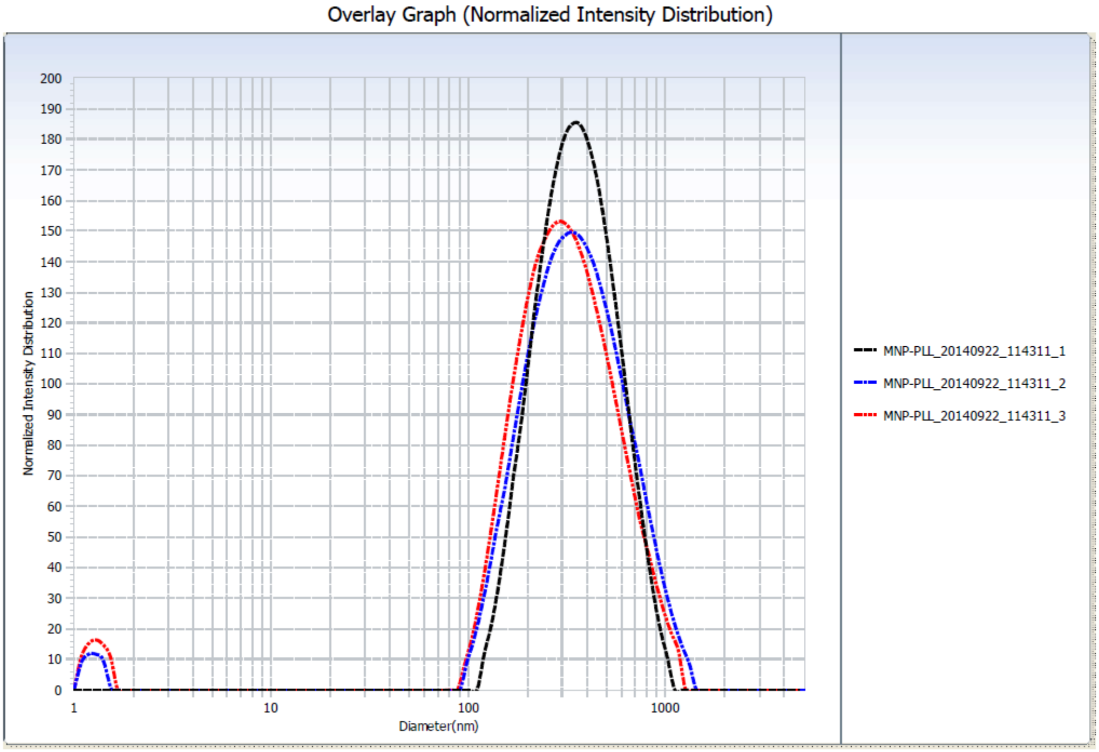

No	Data	Repet. No	pH	Ave. Diameter(nm)	Polydispersity Index	D (10%) (nm)	D (50%) (nm)	D (90%) (nm)
1	MNP-PLL_20140922_114311_1	1	NA	318.0	0.171	183.9	334.8	611.7
2	MNP-PLL_20140922_114311_2	2	NA	300.5	0.235	154.2	324.6	693.0
3	MNP-PLL_20140922_114311_3	3	NA	279.0	0.247	141.8	291.6	625.2

Figure S4: DLS analysis of MNP-PLL samples

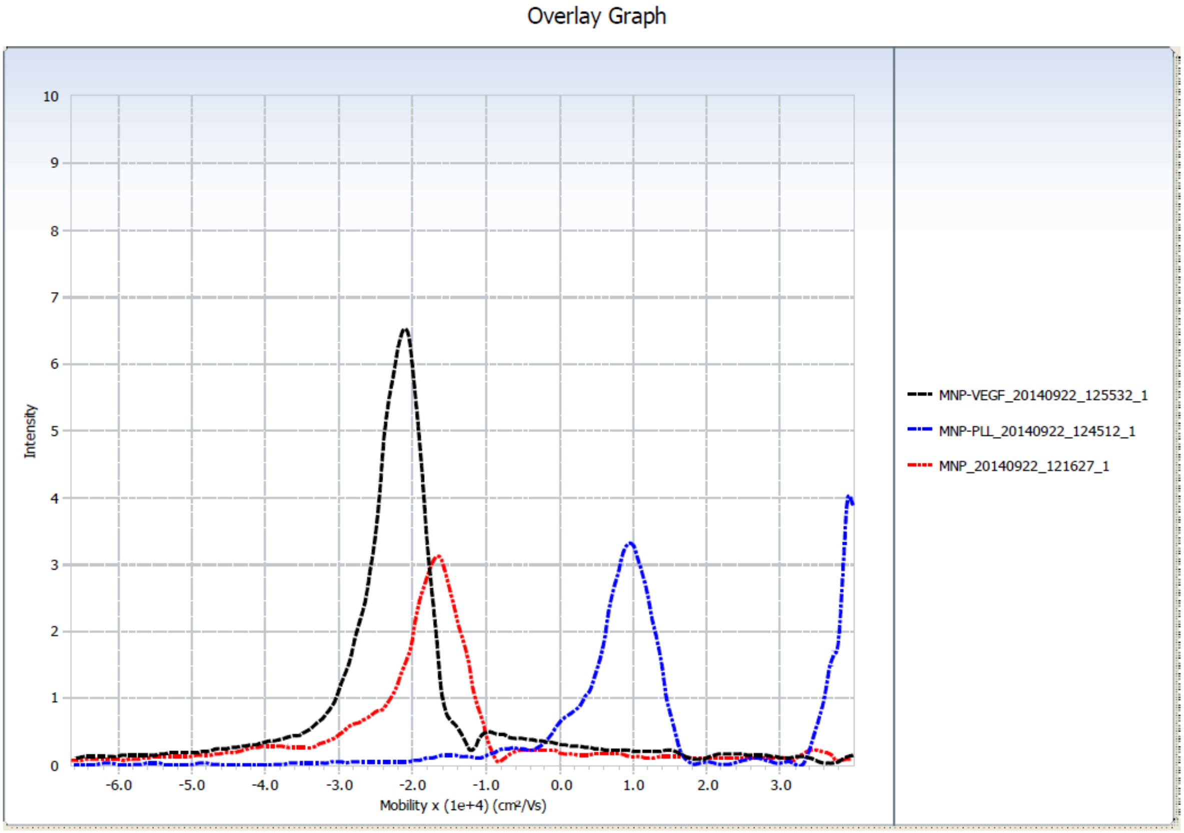

No	File Name	Repet. No	MeasTime	pH	Zeta Potential (mV)	Mobility (cm²/Vs)	E. Field (V/cm)
1	MNP-VEGF_20140922_125532	1	12:55:32	NA	-32.49	-2.164e-004	-77.80
2	MNP-PLL_20140922_124512	1	12:45:12	NA	14.04	9.376e-005	-77.79
3	MNP_20140922_121627	1	12:16:27	NA	-25.59	-1.695e-004	-77.76

Figure S5: Z potential analysis of MNP (red), MNP-rVEGF (black) and MNP-PLL (blue) samples.

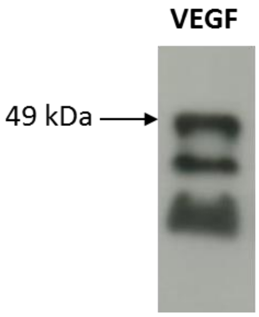

Figure S6: Western blot of purified rVEGF using an antibody against VEGF (sc-507 Santa Cruz, dilution 1:200). The rVEGF has a molecular weight of 49 kDa while the other bands are possibly generated by truncated forms of the fusion protein.

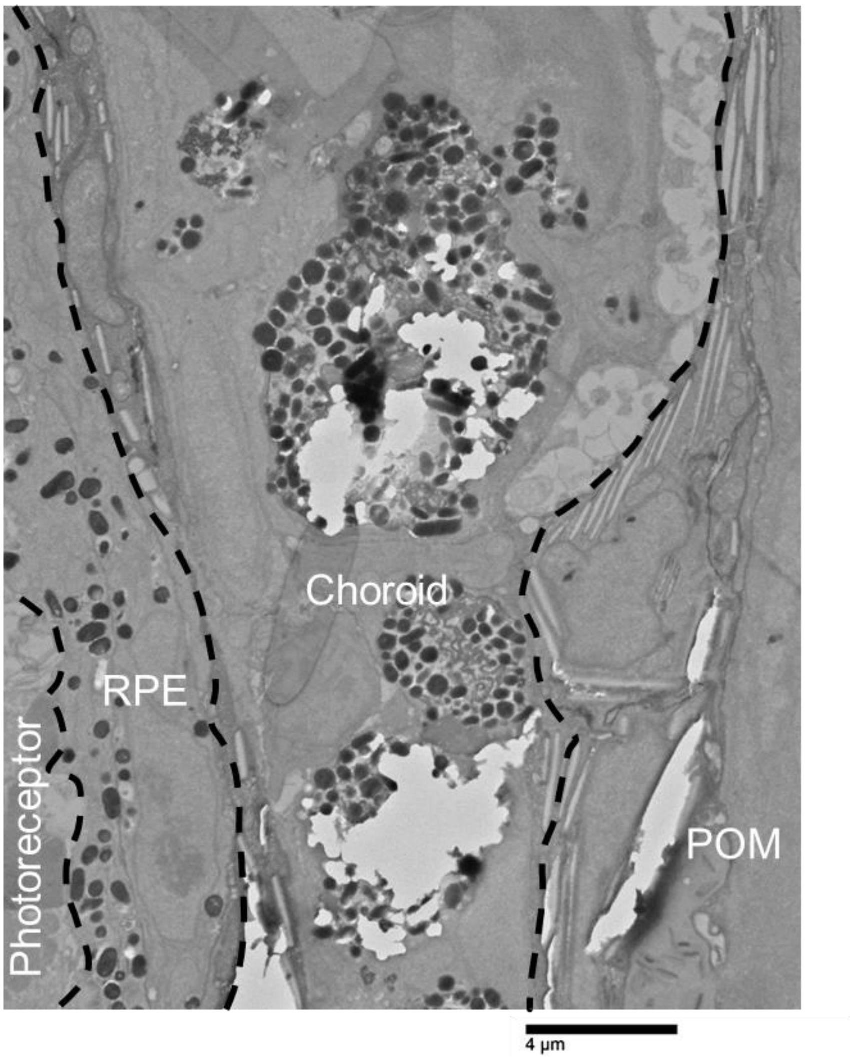

Figure S7: TEM image of an ultrathin section of zebrafish larva eye 72hpf. The photoreceptor, the RPE and the choroid layers are marked in the image. POM: periocular mesenchyme.
